# Detection algorithm for pigmented skin disease based on classifier-level and feature-level fusion

**DOI:** 10.3389/fpubh.2022.1034772

**Published:** 2022-10-20

**Authors:** Li Wan, Zhuang Ai, Jinbo Chen, Qian Jiang, Hongying Chen, Qi Li, Yaping Lu, Liuqing Chen

**Affiliations:** ^1^Dermatology Department, Wuhan No.1 Hospital, Hubei, China; ^2^Dermatology Hospital of Southern Medical University, Guangzhou, China; ^3^Department of Research and Development, Sinopharm Genomics Technology Co., Ltd., Jiangsu, China

**Keywords:** fusion network, pigmented skin disease, attention mechanism, image style transfer, model interpretability

## Abstract

Pigmented skin disease is caused by abnormal melanocyte and melanin production, which can be induced by genetic and environmental factors. It is also common among the various types of skin diseases. The timely and accurate diagnosis of pigmented skin disease is important for reducing mortality. Patients with pigmented dermatosis are generally diagnosed by a dermatologist through dermatoscopy. However, due to the current shortage of experts, this approach cannot meet the needs of the population, so a computer-aided system would help to diagnose skin lesions in remote areas containing insufficient experts. This paper proposes an algorithm based on a fusion network for the detection of pigmented skin disease. First, we preprocess the images in the acquired dataset, and then we perform image flipping and image style transfer to augment the images to alleviate the imbalance between the various categories in the dataset. Finally, two feature-level fusion optimization schemes based on deep features are compared with a classifier-level fusion scheme based on a classification layer to effectively determine the best fusion strategy for satisfying the pigmented skin disease detection requirements. Gradient-weighted Class Activation Mapping (Grad_CAM) and Grad_CAM++ are used for visualization purposes to verify the effectiveness of the proposed fusion network. The results show that compared with those of the traditional detection algorithm for pigmented skin disease, the accuracy and Area Under Curve (AUC) of the method in this paper reach 92.1 and 95.3%, respectively. The evaluation indices are greatly improved, proving the adaptability and accuracy of the proposed method. The proposed method can assist clinicians in screening and diagnosing pigmented skin disease and is suitable for real-world applications.

## 1. Introduction

Skin, as the first layer of protection for the human body, has important physiological protection functions, such as excretion, regulating body temperature and feeling external stimuli. It is also the largest organ in the human body. However, the incidence of skin diseases is extremely high, and there are many types of skin diseases, among which pigmented skin lesions are common; most pathological areas are black, brown or other dark colors, which is mainly due to the increase or decrease in regional melanin caused by ultraviolet radiation or other external factors. In 2021, skin melanoma in pigmented skin disease accounts for 5.6% of all new cancers in the United States, and the number of skin melanoma patients has increased at an annual rate of ~1.4% over the past 10 years (1). However, melanoma that is detected early has a very high cure rate. Studies have shown that if abnormal skin melanocyte proliferation is found early, the survival rate is 96%. If late-stage melanoma is detected, the survival rate is reduced to only 5% (2), and its color is easily confused with that of other common skin pigmented diseases, leading to misdiagnosis. The diagnosis of pigmented skin lesions requires trained specialists, but the number of specialist doctors is grossly inadequate compared to the number of cases. Therefore, it is necessary to develop an algorithm for the automatic diagnosis of pigmented skin lesions.

In recent years, deep learning has been widely used in feature extraction, object classification and detection. Compared with machine learning, deep learning can automatically and efficiently extract features from medical images. Since 2012, various deep Convolutional Neural Network (CNN) models based on the “ImageNet” dataset have been proposed. AlexNet (ImageNet classification with deep convolutional neural networks), a network architecture proposed by Krizhevsky et al. (3), was the winner of the first ImageNet Challenge classification task in 2012; ZFNet (4) (Visualizing and understanding convolutional networks) is a large convolutional network based on AlexNet; VGGNET (5) (Very deep convolutional networks for large-scale image recognition) was proposed by Visual Geometry Group (VGG), a famous research group at Oxford University, and won the first place in localization and the second place in classification in that year's ImageNet competition. GoogleNet (6) (Going deeper with convolutions) was proposed by the Google team and won the first place in the ImageNet competition for the classification task; ResNet (7) (Deep residual learning for image recognition), proposed by Microsoft Research, won the first place in classification task and the first place in target detection in that year's ImageNet competition, and the first place in target detection and image segmentation in COCO dataset. ResNeXt (8) (Aggregated residual transformations for deep neural networks) is a new image classification network proposed by Kaiming He's team at CVPR 2017. ResNeXt is an upgraded version of ResNet; SENET (9) (Squeeze-and-Excitation Networks) is a new image recognition architecture announced by the self-driving company Momenta in 2017. This structure is the first place in the ImageNet competition in that year in the classification task; NASNet (Learning Transferable Architectures for Scalable) is a deep network model proposed by Zoph et al. (10) that can automatically generate network structures without manually designing network models; EfficientNet (11) (EfficientNet: Rethinking model scaling for convolutional neural networks) is proposed by Google team to obtain better performance by deepening the model, widening the model or increasing the resolution of the model input. These network models have ranked highly in competitions. The prediction effects of different network structures in various fields are inconsistent, so researchers cannot quickly find appropriate network models. Many scholars have thus conducted research to solve this problem. Researchers must test the outstanding network models one by one to find the most appropriate network model for their scenario (12–15). This strategy wastes time and resources. Therefore, an ensemble network can obtain an algorithmic model that is better than the model produced by the best individual network by setting the weights of different networks (16–18). However, at present, most network fusion approaches use majority voting, mean voting or the weights of the base classifiers to obtain the output of various networks through one-to-one testing, which cannot give full play to the various effects of different classifiers on different tasks. Therefore, this paper proposes a variety of fusion strategies and optimizes the weight of each classifier through the loss function of the network model to fully utilize the ability of each classifier for the detection of pigmented skin diseases.

Therefore, building a pigmented skin disease detection algorithm based on classifier-level and feature-level fusion encounters the following problems.

(1) How to handle unbalanced pigmented skin disease datasets.
(2) How to build an effective network fusion strategy.

## 2. Related work

In recent years, the applications of Artificial Intelligence (AI) in various fields have developed rapidly, especially in the fields of medical image analysis and bioinformatics. At present, AI is widely used in skin cancer diagnosis (19–21). From the point of view of whether features can be extracted automatically, the AI approaches in this area can be divided into skin cancer classification methods based on machine learning and skin cancer classification methods based on deep learning.

Skin cancer classification based on machine learning generally involves manually extracting image features and then inputting the extracted features into a machine learning algorithm to obtain classification results (22–25). Varalakshmi (26) first used an upsampling method called the Synthetic Minority Oversampling Technique (SMOTE) to balance his dataset, greatly improving the accuracy of various machine learning models. The accuracies of different machine learning algorithms were then analyzed. Support Vector Machine (SVM) algorithms with polynomial kernels provide better accuracy than other machine learning algorithms, such as decision trees using Gini indices and entropy, naive Bayes classifiers, extreme gradient boosting (XGBoost) classifiers, random forests, and logistic regression algorithms. Sabri (19) first extracted the shapes, colors, textures and skeletons of skin image lesions, then used the information gain method to determine the best combination of features, and finally input this feature combination into a commonly used machine learning algorithm to predict the categories of legions. Vidya (27) first extracted skin image asymmetry, border, color, and diameter information. A Histogram of Oriented Gradients (HOG) and a Gray Level Co-occurrence Matrix (GLCM) were used to extract texture features. The extracted features were passed directly to classifiers utilizing different machine learning techniques [such as an SVM, K-Nearest Neighbors (KNN) and a naive Bayes classifier] to classify skin lesions as benign or melanoma. Kalwa (28) presents a smartphone application that combines image capture capabilities with preprocessing and segmentation to extract the Asymmetry, Border irregularity, Color variegation, and Diameter (ABCD) features of a skin lesion. Using the feature sets, classification of malignancy is achieved through support vector machine classifiers.

Skin cancer classification approaches based on deep learning usually adopt a network model for automatic feature extraction, and thus feature extraction and classification can be completed in the same algorithm (20, 21, 29–31). Skin cancer detection algorithms based on deep learning can be divided into single-classifier detection methods and fusion detection methods based on multiple classifiers according to the number of utilized classifiers.

Based on single-classification detection, Sevli (32) proposed using a CNN model to classify seven different skin lesions in the HAM10000 dataset, and the model achieved 91.51% classification accuracy. The model linked its results to a web application and was assessed in two stages by seven dermatologists. Milton (12) first appropriately processed and enhanced skin images and then carried out experiments on various neural networks, including the progressive NASNet (PNASNet)-5-Large, InceptionResNet V2, SENet154, InceptionV4, etc. Finally, the PNASNet-5-Large model achieved the best validation result of 0.76.

Regarding detection based on multiple classifiers, Pal (33) solved the data imbalance problem in the training dataset by setting a propagation-weighted loss from the loss correspondence. For classifier model construction, the pretraining weights of these models were fine-tuned (by ResNet50, DenseNet-121, and MobileNet). Finally, the average category prediction probabilities obtained from these trained networks were used to determine the category labels of the test images. Xie (34) used four pretrained ResNet50 networks to characterize the multiscale information of skin lesions and combined them by using adaptive weighting schemes that could be learned during error propagation. The proposed model achieved an average Area Under Curve (AUC) value of 86.5% on the official ISIC-Skin 2018 validation database. Aldwgeri (35) aimed to solve the data imbalance problem in the training dataset and realized the equalization of each category through flipping, rotation, shifting, and scaling techniques. The equalized image data were then input into different pretraining models, including VGG-Net, ResNet50, Inception V3, Xception, and DenseNet-121. The outputs of the five pretraining models were averaged to produce the final prediction results.

Therefore, the innovations of this paper include the following aspects.

(1) An image style transfer algorithm is applied to the detection of pigmented skin diseases for the purpose of image augmentation.
(2) To prevent image augmentation noise, the required upsampling image is applied to each class image.
(3) Attention mechanisms and common network architectures should be combined to achieve improved detection efficiency.
(4) Two feature-level fusion optimization schemes based on deep features and a classifier-level fusion method based on a classification layer are proposed.
(5) Two visualization algorithms, Grad_CAM and Grad_CAM++, are used to verify the validity of the fusion network.

## 3. Detection algorithm for pigmented skin diseases based on classifier-level and feature-level fusion

### 3.1. System architecture

**Figure 1:**
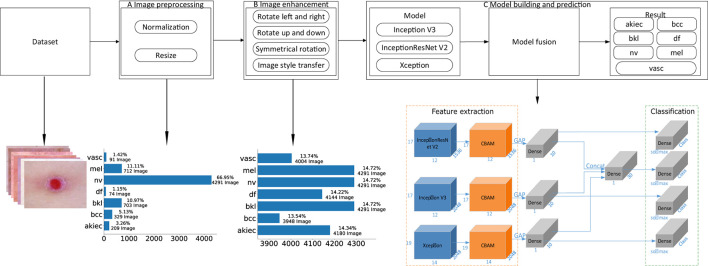
An overview of the proposed method. **(A)** “Image preprocessing,” including image normalization and image resizing, is performed on an input image before feature extraction. **(B)** “Image augmentation,” including operations such as image rotation and image style transfer, performs upsampling on the input image to keep the various categories in a balanced state. **(C)** “Model building and prediction” carries out model training and prediction on the input image, wherein the feature extraction part is the fusion of three base classifiers and an attention mechanism [the convolutional block attention module (“CBAM”)], “GAP” denotes global average pooling, “Dense” is a fully connected layer, “Concat” is the fusion of the output results of the three branches, and “Class” is the number of categories. In this article, Class is 7. “Softmax” is the activation function of the classification output layer, “Classification” is the prediction result output layer, and the number represents the change in the characteristic dimensionality at each stage.

This paper proposes a detection algorithm for pigmented skin diseases based on a fusion network (Figure 1). This approach can be divided into three modules: image preprocessing, image augmentation, and model building and prediction.

Image preprocessing: First, the obtained pigmented skin disease images are normalized, and the pixel values of the images are limited to 0–1, which can effectively reduce the number of calculations required for the images in the neural network. Then, the height and width of each normalized image are unified to 450*600 (*via* nearest-neighbor interpolation). Finally, the preprocessed image dataset (three-channel color images with heights of 450 and widths of 600) for pigmented skin diseases can be obtained. As seen from Figure 1, the proportions of the different categories after image pretreatment are seriously unbalanced; among them, the “nv” category occupies 66.95% of the dataset. If no processing is performed, the neural network will seriously prefer this category in model training.

Image augmentation: As the nv category accounts for 66.95% of the dataset, if dataset balance needs to be achieved, other categories need to be upsampled. First, skin images (except those in the nv category) are preprocessed by turning them left and right, reversing up and down, symmetric rotation (the calculation process is shown in Algorithm 1) and performing image style transfer (the calculation process is shown in Algorithm 2) to achieve a balance between the various categories of images. As seen from Figure 1, the proportion of each category after image augmentation is relatively balanced, accounting for ~14% of the whole dataset of pigmentosa skin disease images.

**Algorithm 1:** Image augmentation—rotation.

Input: Dataset after image preprocessing : *Data*.
Output: training set, validation set, test set.
1: Define the list of stored images after augmentation:*Data*_*train*_*process* = [].
2: The *Data* are divided into a training set *Data*_*train*, a validation set *Data*_*valid* and a test set *Data*_*test* at a 3:1:1 ratio.
3: for *image* → *Data*_*train* **do**
4: if 'image' belongs to category 'nv' **then**
5: Continue.
6: end **if**
7: Add *image* to *Data*_*process*.
8: *Rotates*_*l*_*r* = Rotate *image* left and right.
9: Add *Rotates*_*l*_*r* to *Data*_*train*_*process*.
10: *Rotates*_*u*_*d* = Rotate *image* up and down.
11: Add *Rotates*_*u*_*d* to *Data*_*train*_*process*.
12: *Rotates*_*s* = Rotate *image* Symmetrical.
13: Add *Rotates*_*s* to *Data*_*train*_*process*.
14: end **for**
15: return *Data*_*train*_*process*, *Data*_*valid*, *Data*_*test*.

**Algorithm 2:** Image augmentation—image style transfer.

Input: Training set after image rotation: *Data*_*train*_*process*.
Output: training set *Data*_*train*_*augmentation*.
1: Define the list of stored images after augmentation: *Data*_*train*_*augmentation* = [].
2: Obtain a set of images for each category in *data*_*train*: data_train0, data_train1, data_train2, data_train3, data_train4, data_train5, data_train6.
3: *data*_*train*_*list* = (data_train0, data_train1, data_train2, data_train3, data_train5, data_train6).
4: for *data*_*train*_*i* → *data*_*train*_*list* **do**
5: Calculate the difference between the sample sizes of category *data*_*train*_*i* and category *data*_*train*4 (nv sample): *numSub*.
6: According to *data*_*train*_*i* and *numSub*, calculate the number of images to be upsampled for each category: *numAdd*.
7: if *numAdd* ≥ 1 **then**
8: for *contentImage* → *data*_*train*_*i* **do**
9: *numAdd* images are randomly selected from *data*_*train*_*i*: *styleImageList*.
10: for *styleImage* → *styleImageList* **do**
11: Perform image style transfer using the style image *styleImage* and *contentImage*: *newImage*.
12: Add the image *newImage* to *Data*_*train*_*augmentation*.
13: end **for**
14: end **for**
15: else
16: *numSub* images are randomly selected from *data*_*train*_*i*: *contentImageList*.
17: for *contentImage* → *contentImageList* **do**
18: A images are randomly selected from *data*_*train*_*i*: *StyleImage*.
19: Perform image style transfer using the style image *styleImage* and *contentImage*: *newImage*.
20: Add the image *newImage* to *Data*_*train*_*augmentation*.
21: end **for**
22: end **if**
23: end **for**
24: return *Data*_*train*_*augmentation*.

Model building and prediction: The enhanced images of pigmented skin diseases are first input into three different base classifiers (i.e., Inception V3, InceptionResNet V2, and Xception), and the outputs of the three base classifiers are then fused. Finally, the fusion result is used as the pigmented skin disease prediction result.

### 3.2. Image preprocessing module

#### 3.2.1. Dataset

**Figure 2:**
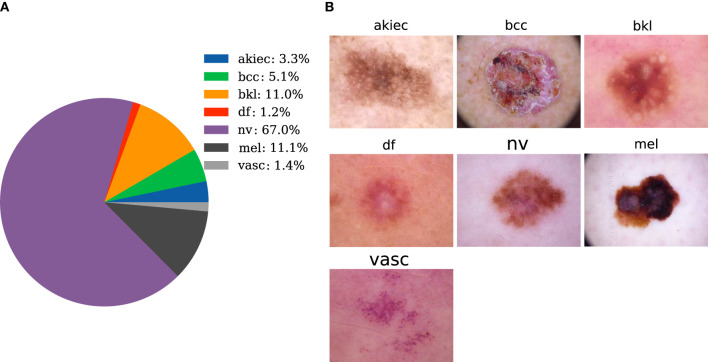
Data preparation. **(A)** The distribution of pigmented skin diseases in the specimens. **(B)** Photos of the representative pigmented skin diseases in each sampling category for clinical diagnosis.

The dataset used in this paper is provided by Tschandl et al. (36), and it contains 10,015 pictures of seven types of skin diseases. Cases include a representative collection of all import diagnostic categories in the realm of pigmented lesions. The seven types are melanocytic Nevi (nv), Melanoma (mel), Benign Keratosis-like Lesions (solar lentigines/seborrheic keratoses and lichen-planus-like keratoses) (bkl), Basal Cell Carcinoma (bcc), Actinic Keratoses and Intraepithelial Carcinoma/Bowen's disease (akiec), Vascular lesions (angiomas, angiokeratomas, pyogenic granulomas, and hemorrhage) (vasc), and Dermatofibroma (df). The corresponding amounts of image data are 6,705, 1,113, 1,099, 514, 327, 142, and 115, respectively. The proportion of each category is shown in Figure 2A. Typical images for each category are shown in Figure 2B. In Figure 2A, the selected dataset of pigmented skin diseases is severely imbalanced between categories, and the imbalance in the dataset causes the model to completely bias the prediction results to the side with a large sample size (18), and the model does not have any prediction effect on the other categories of sample classification, so a processing step for the imbalance in the dataset is necessary.

#### 3.2.2. Image preprocessing and augmentation

**Figure 3:**
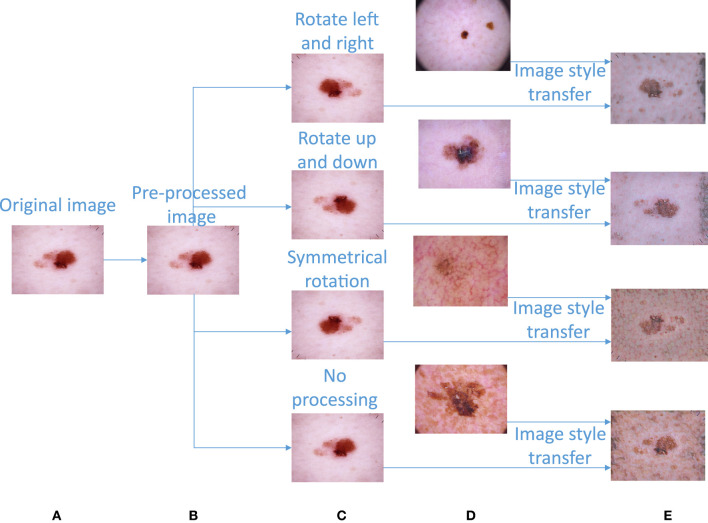
Image preprocessing and image augmentation. **(A)** Original image. **(B)** Preprocessed image. **(C)** Image obtained after a basic rotation operation. **(D)** Image randomly extracted from image category c for image style transfer. **(E)** Composite image obtained after image style transfer.

We first preprocess the acquired skin disease dataset (36) to obtain high-quality image data. In the preprocessing step, each image is first reduced to the specified size of 450*600, and then each pixel of the image is normalized according to Equation (1). In this way, the image is easy for the network to calculate. The image preprocessing part is transformed from Figure 3A to Figure 3B.


(1) $$\begin{array}{l} X_{norm}=\frac{X-X_{min}}{X_{max}-X_{min}} \end{array}$$

The dataset presents great disparities among the amounts of image data contained in various categories. Without performing certain processing steps, the prediction results will be greatly affected by this unbalanced dataset. Therefore, we must upsample the image data to obtain a balanced image dataset. First, we carry out the following basic operations on the images (except for those in the nv category): left and right mirror rotation, up and down mirror rotation, symmetric rotation, etc.; these operations can balance the images to a certain extent. The left and right mirror rotation operations mirror the original image with respect to its vertical centerline. The upper and lower mirror rotation operations mirror the original image with respect to its horizontal centerline. Symmetric rotation is an image transformation that flips the original image left and right before flipping them again in the up and down directions. After completing the basic image operations, the image data contained in different image categories are shown in Table 1. The basic image augmentation operation can be converted from Figure 3B to Figure 3C.

**Table 1:** Image number statistics during image preprocessing.

**Category**	**Number of original images**	**Number of images after basic operations**	**Number of images to be added per image**	**Number of images after image style transfer**
akiec	209	836	4	4,180
bcc	329	1,316	2	3,948
bkl	703	2,812	0.5	4,291
df	74	296	13	4,144
nv	4,291	4,291	0	4,291
mel	712	2,848	0.5	4,291
vasc	91	364	10	4,004

It can be seen from Table 1 that the numbers of images in various categories are still seriously imbalanced, so we adopt an image style transfer algorithm (37) to upsample the images. The image style transfer algorithm proposed by Ghiasi has been successfully trained on a corpus of ~80,000 paintings. In addition, it can be generalized to previously unobserved images.

First, this paper calculates the sample size differences between nv and the other categories in the image dataset according to Equation (2) and then divides each difference by the sample size of the corresponding category to obtain the sample size “n” that needs to be randomly added to the other categories. The image to be upsampled is selected as the “content image,” “*n*” images are randomly selected from the image samples of this category as the “style images,” and the “content image” and “*n*” “style images” are input into the image style transfer model in turn to obtain “*n*” upsampling images generated by the fusion of the “content image” and “style images” (the calculation process is shown in Algorithm 2). After performing image style transfer, the amount of data in each category is shown in Table 1. An example diagram of image style transfer is shown in Figures 3C–E.


(2) $$\begin{array}{l} Add_{n}=\frac{Num\left(Class\text{\_}nv\right)-Num\left(Class\text{\_}i\right)}{Num\left(Class\text{\_}i\right)} \end{array}$$

In the equation, i represents the akiec, bcc, bkl, df, mel, and vasc categories; Num(Class_i) represents the data volume of the selected category. If *Add*_*n*_ is <1, it indicates that the data volume of this category is not very different from that of nv. In this paper, the number of data differences is randomly extracted for image style transfer.

### 3.3. Model building and prediction module

The base classifier of the fusion network used in this paper can consist of Inception V3, InceptionResNet V2, and Xception. The fusion part explores feature-level fusion based on deep features and classifier-level fusion based on a classification layer.

Feature-level fusion based on deep features has been proven to be an efficient fusion strategy (38–42) that can combine features extracted from N networks into a single feature vector containing more image information. Feature-level fusion techniques can be divided into parallel feature-level fusion and serial feature-level fusion based on whether the feature dimensions output by the networks are consistent. Three methods are available for realizing parallel feature fusion: summing up each feature (Equation 3); averaging each feature (Equation 4); and executing the max operation (Equation 5) for each feature. Serial feature fusion can only realize feature splicing (Equation 6) according to the channel dimension because of the inconsistency of the feature output dimensions. The classifier-level fusion method based on a classification layer can make the features extracted from *N* networks remain unchanged and perform feature splicing at the output of the classification layer.


(3) $$\begin{array}{l} F_{feature\text{\_}level\text{\_}fusion}=\sum_{i=1}^{N}F_{i} \end{array}$$

(4) $$\begin{array}{l} F_{feature\text{\_}level\text{\_}fusion}=\frac{1}{N}\sum_{i=1}^{N}F_{i} \end{array}$$

(5) $$\begin{array}{l} F_{feature\text{\_}level\text{\_}fusion}=max\left(F_{1},F_{2},F_{3},...,F_{N}\right) \end{array}$$

(6) $$\begin{array}{l} F_{Decision\text{\_}level\text{\_}fusion}=Concat\left(F_{1},F_{2},F_{3},...,F_{N}\right) \end{array}$$

**Figure 4:**
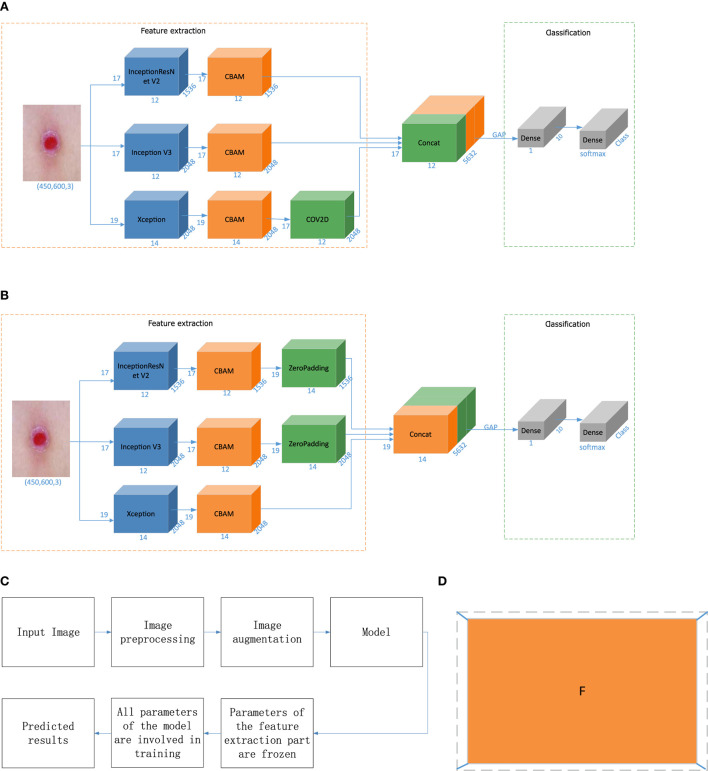
Model building and prediction. **(A)** Fusion based on the convolution operation. **(B)** Fusion based on the padding operation. **(C)** Model training process. **(D)** Zero-padding operation. “CBAM” is the attention mechanism, “zero padding” involves filling a circle of 0 s around the height and width of the feature vector, “Concat” denotes feature fusion, “GAP” is a global average pooling layer, “Dense” is a fully connected layer, “COV2D” is a convolution operation, and “Class” is the number of categories. In this article, Class is 7. “Softmax” is the activation function of the classification output layer, and the number represents the change in the dimensions in each stage.

When the input picture size is (Batch, 450, 600, 3), the output dimensions of Inception V3 are (Batch, 12, 17, 2048), the output dimensions of InceptionResNet V2 are (Batch, 12, 17, 1536), and the output dimensions of Xception are (Batch, 14, 19, 2048). In this paper, feature-level fusion based on deep features employs the output fusion results of three different networks, and the dimensions of the outputs of the three models are inconsistent. Therefore, we optimize the feature-level fusion strategy based on deep features. In the first method, the convolution layer is used to convert the feature map to achieve dimensional consistency. The dimension conversion method is shown in Equations (7) and (8), and the overall algorithm flow is shown in Figure 4A.


(7) $$\begin{array}{l} W_{out}=\frac{W_{in}-F+2P}{S}+1 \end{array}$$

(8) $$\begin{array}{l} H_{out}=\frac{H_{in}-F+2P}{S}+1 \end{array}$$

In the equation, *W*_*in*_ and *H*_*in*_ are the width and height of the input, F is the size of the filter, P is the padding size, S is the step size, and *W*_*out*_ and *H*_*out*_ are the final width and height, respectively. *W*_*in*_ and *H*_*in*_ are 14 and 19, and *W*_*out*_ and *H*_*out*_ are 12 and 17, respectively. Therefore, according to this equation, we set F as 3, P as 0, and S as 1. The output can realize the splicing of the three dimensions.

In the second method, in this paper, the outputs of Inception V3 and InceptionResNet V2 are surrounded by a circle of 0s to achieve dimensionality consistency with Xception. The zero-padding operation is shown in Figure 4D. The fusion process is shown in Figure 4B.

Classifier-level fusion is performed based on the classification layer. This paper first fuses the last convolution layer of each of the three different networks with the Convolutional Block Attention Module (CBAM), then performs global average pooling on this basis, splices a fully connected layer to obtain the final feature vector, and performs a simple splicing operation on the three feature vectors. Finally, the splicing result is input into the classification layer to output the final predicted category value, as shown in Figure 1 in the model building stage. In this way, the network outputs four values corresponding to Inception V3, InceptionResNet V2, Xception, and a merged output. The loss value of the network is the sum of the loss values of the four parts, but the final output is the overall output of the network.

In Figures 1, 4, “CBAM” is an attention mechanism proposed by Woo (43) in 2018. Woo applied attention to both the channel and spatial dimensions. Similar to the SENet[10], a CBAM can be embedded in most mainstream networks at present. The feature extraction capability of a network model can be improved without significantly increasing its computational complexity and number of parameters. Therefore, this paper embeds a CBAM into the feature extraction part to improve the feature extraction ability of the model and facilitate the subsequent network classification ability improvement.

Transfer learning transfers knowledge learned from a source dataset to a target dataset. Fine-tuning is a common technique for transfer learning. The target model replicates all the model designs and their parameters on the source model except the output layer, and fine-tunes these parameters based on the target dataset. The output layer of the target model, on the other hand, needs to be trained from scratch. The whole process of model building and prediction is shown in Figure 4C. First, all the parameters of the base classifier are “frozen” to prevent large planned changes in these parameters during the initial network training. Subsequently all parameters of the network model are “unfrozen” and the parameters of the entire network are fine-tuned to achieve classification of skin diseases.

## 4. Experiment

### 4.1. Experimental conditions

The experimental environment includes Linux X86_64, an Nvidia Tesla V100, and 16 GB of memory. This experiment is based on Python version 3.7.9, TensorFlow version 2.3.0, and Keras version 2.4.3.

### 4.2. Evaluation criteria

In this study, the accuracy, recall, specificity, precision, F1, weighted AUC and AUC metrics are used to evaluate pigmented skin disease detection methods based on a fusion network. The model evaluation confusion matrix and calculation equations are shown in Table 2, respectively.

**Table 2:** Evaluation criteria.

**Evaluation criteria**	**Equation**	**Meaning**
Accuracy (Acc)	$\frac{TP+TN}{TN+FP+FN+TP}$	The proportion of all results correctly judged by the classification model to the total sample size
Sensitivity=Recall	$\frac{TP}{TP+FN}$	The proportion of correct model predictions among all the results whose true values are positive
Specificity	$\frac{TN}{TN+FP}$	The proportion of correct model predictions among all the results whose true values are negative
Precision	$\frac{TP}{TP+FP}$	The proportion of correct model predictions among all the results for which the predicted value of the model is positive
F1	$\frac{2*Precision*Recall}{Precision+Recall}$	Harmonic mean of precision and recall
Weighted avg	$\frac{\overset{class\text{\_}num}{\underset{i=1}{\sum }}P\text{\_}i*support\text{\_}i}{\overset{class\text{\_}num}{\underset{i=1}{\sum }}support\text{\_}i}$	The weighted average of evaluation indicators for each category, with the weight being the proportion of the sample size of each category in the total sample size. “support_i” represents the number of samples in category “i,” “P_i” represents the score value of the evaluation index of category “i,” and “class_num” represents the number of categories.
AUC		Area under the receiver operating characteristic (ROC) curve

True Negatives (TNs) represent the number of cases for which the real values are negative and the model thinks they are negative.

False Positives (FPs) represent the number of cases for which the real values are negative and the model thinks they are positive.

False Negatives (FNs) represent the number of cases for which the real values are positive and the model thinks they are negative.

True Positives (TPs) represent the number of cases for which the real values are positive and the model thinks they are positive.

### 4.3. Determination of the experimental parameters

#### 4.3.1. Test results of a single classifier

In this paper, Inception V3 and cbam fusion are used to test three data augmentation methods. The first (column 4 of Table 3) class weights are calculated by adjusting the model to include a penalty for prediction error for classes with smaller sample sizes, and the weight parameters for each class are calculated as follows.


(9) $$\begin{array}{l} Weight=\frac{n\text{\_}samples}{n\text{\_}classes*bincount\left(y\right)} \end{array}$$

Where *n*_*samples* represents the total number of picture samples,*n*_*classes* represents the number of categories, and *bincount*(*y*) represents the sample size of each category in the training set. Weight is the weight corresponding to each category. The lower the sample size of the category, the higher its weight.

**Table 3:** The influence of the data imbalance treatment scheme on the results.

**Data imbalance processing**		**Original set**	**Class weight**	**Image rotation**	**Pixel**
Precision	akiec	0.6515	0.557	0.7368	0.7167
	bcc	0.7364	0.6056	0.787	0.8333
	bkl	0.7014	0.7125	0.8144	0.7991
	df	0.7692	0.5714	1	0.8
	nv	0.8957	0.8801	0.8877	0.9155
	mel	0.7083	0.6589	0.7517	0.7861
	vasc	0.6	0.7037	0.75	0.8077
	**Weighted avg**	**0.8318**	**0.8064**	**0.8538**	**0.8748**
Recall	akiec	0.6615	0.6615	0.6462	0.6615
	bcc	0.7864	0.8252	0.8252	0.8252
	bkl	0.6727	0.7773	0.7182	0.7773
	df	0.4348	0.6957	0.4348	0.6957
	nv	0.9545	0.9612	0.9672	0.9612
	mel	0.4574	0.6099	0.4888	0.6099
	vasc	0.6429	0.75	0.75	0.75
	**Weighted avg**	**0.8397**	**0.8168**	**0.8597**	**0.8792**
F1	akiec	0.6565	0.688	0.6885	0.688
	bcc	0.7606	0.8293	0.8057	0.8293
	bkl	0.6868	0.788	0.7633	0.788
	df	0.5556	0.7442	0.6061	0.7442
	nv	0.9242	0.9378	0.9258	0.9378
	mel	0.5559	0.6869	0.5924	0.6869
	vasc	0.6207	0.7778	0.75	0.7778
	**Weighted avg**	**0.8315**	**0.8023**	**0.8508**	**0.8753**

The second uses image flipping (column 5 of Table 3) to flip the category with a small sample size to flip the image left and right, invert it up and down, and flip it systematically so that the imbalance between its various categories is somewhat mitigated.

For the network model, a change in a pixel value of an image represents that this image will then change. Therefore, the third one (column 6 of Table 3) is based on the second one to achieve a complete balance between its various categories. The interval of increasing and decreasing pixel values is first calculated by the equation, and then a random value is randomly drawn from the interval without put-back as the increasing or decreasing pixel value.


(10) $$\begin{array}{l} Pixel=\frac{differences}{2*n\text{\_}classes} \end{array}$$

Where *n*_*classes* represents the number of categories, and *differences* represents the difference between this category and the category “nv.” Therefore, the interval of image increase and decrease is from 1 to Pixel.

From Table 3, it can be seen that the effect of solving the data imbalance by changing the calculation method of the model loss values decreases the correct prediction rate compared to the dataset without any change, mainly because the change of the loss values causes the model to have some bias between the categories during training. By changing the image flip compared to not making any changes, the imbalance between categories is somewhat alleviated, so the prediction accuracy is somewhat improved, but there is still some imbalance between categories. Based on the image flip, each image is randomly added or subtracted a certain pixel value to get a brand new image, thus achieving a balance between each category of the image and a certain improvement in prediction.

Therefore, in this paper, we use the image style transfer upsampling scheme to equalize the dataset. After completing dataset equalization, in the single-classifier experiment, we successively change the model module in Figure 4C into three algorithm models: “Inception V3+CBAM,” “InceptionResNet V2+CBAM,” “Xception,” and “Xception+CBAM.” The algorithm test results are shown in Table 4. It can be seen from the third to the sixth column of Table 3 and the third column of Table 4 that the effects of the original dataset, image preprocessing, pixel change and image style transfer on the detection of pigmented skin lesions based on Inception V3 are improved in order, and the accuracy of image style transfer regarding the detection of pigmented skin lesions is 4% higher than that of image preprocessing. It is proven that image style transfer is effective for the detection of pigmented skin lesions. From column 5 and column 6 of Table 4, it can be seen that the presence or absence of the attention mechanism makes some difference to the classification effect (Acc, F1, Specificity), thus proving the contribution of the attention mechanism in the classification of pigmented skin diseases. However, it can be seen from the Acc and F1 values in the table that the detection rate of the “nv” category is much higher than that of the other categories, indicating that a single model has certain anti-interference ability limitations with respect to the images generated by the algorithm.

**Table 4:** The influence of a single network model on evaluation metrics.

**Single algorithm**		**Inception V3**	**InceptionResNet**	**Xception_No_CBAM**	**Xception**
Acc	akiec	0.7538	0.6769	0.6923	0.7077
	bcc	0.8932	0.9223	0.8544	0.8835
	bkl	0.8045	0.7955	0.8091	0.8182
	df	0.7391	0.6522	0.8696	0.7391
	**nv**	**0.9679**	**0.9791**	**0.9754**	**0.9724**
	mel	0.6771	0.5964	0.6233	0.6682
	vasc	0.8928	0.8929	0.8929	0.8929
	**Weighted avg**	**0.9031**	**0.8987**	**0.9002**	**0.9046**
F1	akiec	0.7597	0.7273	0.7563	0.7541
	bcc	0.8762	0.9223	0.8756	0.8545
	bkl	0.8290	0.8140	0.8109	0.8353
	df	0.8293	0.7317	0.8696	0.8095
	**nv**	**0.9495**	**0.9460**	**0.9482**	**0.9525**
	mel	0.7438	0.7056	0.7221	0.7358
	vasc	0.8475	0.9091	0.8772	0.9091
	**Weighted avg**	**0.9007**	**0.8934**	**0.8961**	**0.9018**
Specificity	akiec	0.9923	0.9938	0.9954	0.9943
	bcc	0.9921	0.9958	0.9947	0.9900
	bkl	0.9832	0.9804	0.977	0.9826
	df	0.9995	0.9985	0.9985	0.9990
	**nv**	**0.8565**	**0.8157**	**0.8338**	**0.8595**
	mel	0.9820	0.9882	0.9871	0.9815
	vasu	0.9970	0.9990	0.9978	0.9990
	**Weighted avg**	**0.8994**	**0.8727**	**0.8843**	**0.9012**
AUC	akiec	0.9832	0.9805	0.9835	0.9843
	bcc	0.9938	0.9976	0.9963	0.9954
	bkl	0.9787	0.9769	0.9835	0.9813
	df	0.9910	0.9971	0.9928	0.9959
	nv	0.9802	0.9800	0.9806	0.9775
	mel	0.9651	0.9643	0.9599	0.9613
	vasc	0.9772	0.9930	0.9988	0.9870
	**Weighted avg**	**0.9792**	**0.9792**	**0.9799**	**0.9776**

#### 4.3.2. Fusion test results of multiple classifiers

The detection effect of multinetwork fusion can generally strengthen the generalization ability of a model, thereby improving its detection ability. After performing dataset equalization, we first compare different fusion methods in terms of their final classification effects in multiple classifier experiments, and we test the feature-level fusion approach based on deep features and the classifier-level fusion method based on the classification layer. All three fusion strategies use Inception V3, InceptionResNet V2, and Xception as the three base classifiers. The first feature-level fusion method based on deep features reduces the dimensionality of a feature graph with a larger output through the convolution layer to realize the splicing of dimensions. The second feature-level fusion method based on deep features adds feature graphs with smaller output dimensions to larger feature graphs with the zero-padding operation. The third classifier-level fusion method based on the classification layer splices the outputs of the fully connected layers of the three base classifiers.

Three kinds of fusion strategy evaluation indices are shown in Table 5. According to the data supplied by the convolution layer, the first one-dimensional characteristic figure of dimensionality reduction is generally low. The main reason for this is that adding a convolution layer results in many parameters that need to be trained. The first network loss value is large and can lead to difficult network training for reaching a more appropriate stage. As a result, the overall parameters of the network cannot achieve good results. If zero padding is used, the small-dimensional feature graph is extended, and no redundant parameter training requirement is imposed. Therefore, the output result will be consistent with the transfer learning result. The third method is to splice the output of the fully connected layer, and the final prediction index is the best option. First, the feature extraction part of the network contains the network parameters trained by ImageNet, and the features are relatively appropriate. Finally, only the parameters of the fully connected layer are added; thus, the feature extraction process of the network model does not change, and the final prediction effect is also the best.

**Table 5:** The influence of different fusion strategies on evaluation metrics.

**Fusion network**		**Concat_Conv2D**	**Concat_Zeropadding**	**Concat_Dense**
Acc	akiec	0.7538	0.6923	0.8154
	bcc	0.8058	0.8641	0.9417
	bkl	0.7955	0.8500	0.8409
	df	0.4348	0.6957	0.8261
	nv	0.9418	0.9612	0.9828
	mel	0.6682	0.6323	0.6502
	vasc	0.8929	0.8571	0.9286
	**Weighted avg**	**0.8757**	**0.8942**	**0.9201**
F1	akiec	0.7424	0.7258	0.7737
	bcc	0.8342	0.8812	0.9372
	bkl	0.7743	0.8184	0.8768
	df	0.5882	0.7619	0.8837
	nv	0.9383	0.9467	0.9572
	mel	0.6882	0.7050	0.7532
	vasc	0.7812	0.8276	0.8966
	**Weighted avg**	**0.8745**	**0.8914**	**0.9170**
Specificity	akiec	0.9907	0.9928	0.9902
	bcc	0.9932	0.9947	0.9963
	bkl	0.9680	0.9720	0.9905
	df	0.9995	0.9985	0.9995
	nv	0.8671	0.8595	0.8565
	mel	0.9657	0.9798	0.9904
	vasc	0.9944	0.9970	0.9980
	**Weighted avg**	**0.9029**	**0.9001**	**0.9013**
AUC	akiec	0.9706	0.9839	0.9912
	bcc	0.9801	0.9955	0.9984
	bkl	0.9651	0.9754	0.9899
	df	0.9589	0.9821	0.9923
	nv	0.9653	0.9741	0.9853
	mel	0.9227	0.9514	0.9722
	vasc	0.9728	0.9890	0.9765
	**Weighted avg**	**0.9615**	**0.9734**	**0.9852**

From the weighted average of the Acc and F1 values in Tables 4, 5, it can be seen that the model training and prediction steps performed by a single classifier are better than those of the two fusion strategies based on feature-level fusion. The main reason for this involves the changes in the extracted image features during feature-level fusion. Compared with the better network feature extraction ability of “ImageNet” training, the feature extraction ability of the modified network exhibits a certain decline, resulting in a decrease in the classification index based on feature-level fusion. During feature extraction, the classifier-based fusion scheme does not change the feature extraction capability of the original network based on “ImageNet.” Features are learned separately through the convolution layer of each base classifier, and the results of the fully connected network (i.e., the classifier) of the base classifier are fused to obtain the final predicted category value. Based on classifier-level fusion, the output results of multiple base classifiers are fused. The generalization ability and anti-interference ability of the network are enhanced, and the model classification ability is enhanced.

#### 4.3.3. Setting the number of fusion networks

**Figure 5:**
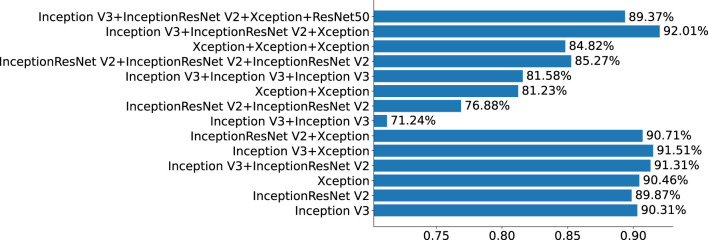
Effect of the number of classifiers on the resulting network.

This section mainly studies how to combine base classifiers in fusion networks to achieve the best effect for the detection of pigmented skin lesions. This paper mainly tests the effectiveness of combinations including three basic classifiers: Inception V3, InceptionResNet V2, and Xception. The fusion effects of two networks, three networks, four networks, etc. are tested. The best fusion scheme (classifier-level fusion based on the classification layer in Section 4.3.2) is adopted. Six scenarios are available regarding the fusion of two networks, as shown in the table: fusing Inception V3 with Inception V3, InceptionResNet V2 with InceptionResNet V2, Xception with Xception, Inception V3 with InceptionResNet V2, Inception V3 with Xception, and InceptionResNet V2 with Xception. Four scenarios are considered regarding the fusion of three networks, as shown in the table: the fusion of Inception V3, Inception V3, and Inception V3; the fusion of InceptionResNet V2, InceptionResNet V2, and InceptionResNet V2; the fusion of Xception, Xception, and Xception; and the fusion of Inception-V3, Inception-ResNet-V2, and Xception. The four-network case is a fusion of Inception V3, InceptionResNet V2, Xception, and ResNet50. It can be seen from Table 6 and Figure 5 that if two base classifiers are consistent in the fusion process of two networks, the classification effect will be worse than that of using one base classifier alone. In a fusion network, there must be some difference between the base classifiers; otherwise, the network easily falls into local minima during the training process. It can be seen from Table 6 that when two different base classifiers are used, the classification accuracy is greatly improved compared with that of a network containing two identical classifiers. From the values listed in Table 6, the monitoring indices of Inception V3_InceptionResNet, Inception V3_Xception, and Inception V3_InceptionResNet are better than those of single Inception V3, InceptionResNet, Xception models; It can be seen from the data in Table 7 that the fusion effect of four networks is not as good as that of three networks, thus proving that the network fusion does not guarantee that a greater number of base classifiers leads to better results. Therefore, the fusion method based on Inception V3, InceptionResNet V2, and Xception is finally selected as the network model in this paper.

**Table 6:** The influence of fusion of two base classifiers on evaluation metrics.

**Two network**		**Inception V3_Inception V3**	**InceptionRes Net_Inception-ResNet**	**Xception_ Xception**	**Inception V3_Inception-ResNet**	**Inception V3_Xception**	**InceptionRes-Net_Xception**
Acc	akiec	0.2462	0.4923	0.5231	0.7692	0.7231	0.7538
	bcc	0.7282	0.7087	0.7864	0.8932	0.9223	0.9417
	bkl	0.5682	0.6364	0.6318	0.8500	0.8455	0.8409
	df	0.4783	0.5217	0.3913	0.7391	0.8696	0.7391
	nv	0.8069	0.8673	0.9314	0.9851	0.9761	0.9754
	mel	0.4439	0.4350	0.4036	0.6143	0.6726	0.6054
	vasc	0.6786	0.8214	0.8929	0.8929	0.9286	0.9286
	**Weighted avg**	**0.7124**	**0.7688**	**0.8123**	**0.9131**	**0.9151**	**0.9071**
F1	akiec	0.3596	0.5289	0.5574	0.7937	0.7520	0.7967
	bcc	0.6198	0.6759	0.7364	0.8846	0.8962	0.9194
	bkl	0.4505	0.5501	0.6347	0.8539	0.8493	0.8565
	df	0.4889	0.5581	0.5143	0.8095	0.9091	0.8293
	nv	0.8547	0.8851	0.9021	0.9555	0.9583	0.9471
	mel	0.4033	0.4491	0.4932	0.7366	0.7557	0.7124
	vasc	0.7308	0.8070	0.8772	0.9091	0.8966	0.8966
	**Weighted avg**	**0.7259**	**0.7726**	**0.8027**	**0.9088**	**0.9124**	**0.9027**
Specificity	akiec	0.9959	0.9943	0.9881	0.9943	0.9933	0.9954
	bcc	0.9663	0.9932	0.9811	0.9932	0.9926	0.9942
	bkl	0.8822	0.9826	0.9557	0.9826	0.9821	0.9849
	df	0.9944	0.9990	0.9985	0.9990	0.9995	0.9995
	nv	0.8353	0.8444	0.7296	0.8444	0.8761	0.8293
	mel	0.9051	0.9933	0.9708	0.9933	0.9865	0.9882
	vasc	0.9975	0.9990	0.9980	0.9990	0.9980	0.9980
	**Weighted avg**	**0.8643**	**0.8926**	**0.8094**	**0.8926**	**0.9130**	**0.8823**
AUC	akiec	0.946	0.9509	0.9427	0.9793	0.9807	0.9909
	bcc	0.9585	0.9772	0.9855	0.9976	0.9972	0.9977
	bkl	0.8584	0.9	0.9304	0.9849	0.9798	0.9836
	df	0.9315	0.9436	0.974	0.9975	0.9966	0.9937
	nv	0.9147	0.9239	0.9396	0.9819	0.9837	0.9818
	mel	0.8499	0.8645	0.9046	0.9682	0.9708	0.9633
	vasc	0.9920	0.9959	0.9963	0.9823	0.9921	0.9868
	**Weighted avg**	**0.9058**	**0.9195**	**0.9383**	**0.9816**	**0.9827**	**0.9813**

**Table 7:** The influence of fusion of multiple base classifiers on evaluation metrics.

**Multi-network fusion**		**Inception V3_Inception V3_Inception V3**	**InceptionResNet_ InceptionResNet_ InceptionResNet**	**Xception_ Xception_ Xception**	**Inception V3_ InceptionResNet_ Xception_ ResNet50**
Acc	akiec	0.6308	0.7385	0.7077	0.7846
	bcc	0.7961	0.7573	0.8058	0.8738
	bkl	0.7045	0.7318	0.7227	0.8455
	df	0.4783	0.4783	0.4348	0.7391
	nv	0.9150	0.9493	0.9493	0.9679
	mel	0.4170	0.5112	0.4709	0.5561
	vasc	0.8929	0.8214	0.8214	0.8571
	**Weighted avg**	**0.8158**	**0.8527**	**0.8482**	**0.8937**
F1	akiec	0.5857	0.7164	0.6765	0.7286
	bcc	0.7421	0.7464	0.8098	0.8738
	bkl	0.6610	0.7523	0.7413	0.8176
	df	0.6111	0.6111	0.5882	0.8500
	nv	0.9065	0.9228	0.9158	0.9492
	mel	0.4987	0.5891	0.5707	0.6667
	vasc	0.8333	0.8679	0.8519	0.8276
	**Weighted avg**	**0.8109**	**0.8468**	**0.8403**	**0.8894**
Specificity	akiec	0.9825	0.9892	0.9871	0.8860
	bcc	0.9811	0.9853	0.9900	0.9330
	bkl	0.9473	0.9736	0.9720	0.9090
	df	0.9990	0.9990	0.9995	0.8700
	nv	0.7900	0.7810	0.7492	0.9110
	mel	0.9680	0.9719	0.9775	0.7710
	vasc	0.9965	0.9990	0.9985	0.9270
	**Weighted avg**	**0.8485**	**0.8462**	**0.8256**	**0.8953**
AUC	akiec	0.9511	0.9769	0.9803	0.9866
	bcc	0.9820	0.9895	0.9900	0.9964
	bkl	0.9309	0.9650	0.9611	0.9726
	df	0.9505	0.9656	0.9826	0.9821
	nv	0.9413	0.9611	0.9495	0.9754
	mel	0.9070	0.9426	0.9082	0.9591
	vasc	0.9838	0.9993	0.9894	0.9971
	**Weighted avg**	**0.9394**	**0.9620**	**0.9502**	**0.9751**

To explore the performance of different network combinations in the feature extraction framework, we perform ablation experiments for each image classification configuration. The first case utilizes combinations with the same subnetwork. With the increase in the number of networks (columns 3, 4, 6 in Table 4, 3–5 in Table 6, and 3–5 in Table 7), the classification performance declines. Therefore, it is not better to increase the number of subnetwork when they are the same. The possible reason for this finding is that overfitting easily occurs in overly complex networks, which leads to performance degradation. However, the classification performance shown in Table 7 is higher than that in Table 6. The main reason for this is that in ensemble learning, the number of general base classifiers cannot appear to be even; otherwise, the same predicted value is likely to occur, and random judgment may occur during model classification. The second was for different subnetworks. With the increase in the number of networks (columns 3, 4, 6 in Table 4, columns 6–8 in Table 6, and columns 6 in Table 7), the classification performance increases first and then decreases, indicating that increasing the number of subnetworks can improve the accuracy of pigmented skin lesion detection, but more is not always better. The overfitting of complex networks may also occur. Third, it can be seen from Table 6 that when the number of networks is the same, the performance obtained when using different subnetworks as feature extractors is better than that achieved with identical subnetworks. These results prove the feasibility of the proposed network.

### 4.4. Comparison of the experimental results obtained by the proposed methods

According to the test results, the comparison between this study and similar recent studies is shown in Table 8. The dataset listed in Table 8 is HAM10000, which was presented in the ISIC 2018 Challenge and is used in this study. From the evaluation indices obtained on the test set, it can be seen that the data upsampling scheme based on image flipping and image style transfer proposed in this paper can produce the same amount of data in each category; In addition, network fusion schemes based on available data can achieve higher detection efficiency for pigmented skin lesions than hard voting fusion schemes.

**Table 8:** Comparison of the results obtained in this study with those in the literature.

**References**	**Method**	**Results**
Sevli (32)	Custom CNN model	The accuracy on test set reaches 91.51%
Salian et al. (44)	Custom CNN model	The test accuracy is 83.15%
Pal et al. (33)	Ensemble (ResNet50, DenseNet-121, and MobileNet)	The normalized multiclass accuracy is 77.5%
Xie et al. (8)	multilevel deep ensemble (MLDE) model	The result is an average AUC of 86.5
Aldwgeri and Abubacker (35)	Ensemble[VGG, ResNet50, Inception-V3, Xception, and DenseNet-121]	Multiclass accuracy of 80.1% and mean average of 0.89 AUROC
Hard voting	Ensemble (Inception V3, InceptionResNet V2, and Xception)	The accuracy on test set reaches 91.61%
Proposed fusion network	Fusion network (Inception V3, InceptionResNet V2, and Xception)	The accuracy and AUC on the test set reach 92.01 and 95.3%, respectively

### 4.5. Experimental expansion

In order to validate the impact of the developed fusion network on external test data, the UCSD common retinal OCT dataset (45) was collected with a total sample size of 108,309 images in four categories: Normal, Drusen, CNV, and DME. The sample sizes of the four categories are 51,140, 8,616, 37,205, and 11,348, respectively, and this paper focuses on the “limited model,” i.e., 1,000 randomly selected images in each category, to compare the performance using the fusion strategies. Table 9 shows that the overall accuracies of the three fusion strategies are 97.4, 97.5, and 98.7%, respectively. Compared with the model proposed by Kermany (46), the accuracy is 93.4%, which is an average improvement of 4% points. Overall, the three fusion strategies proposed in this paper are effective.

**Table 9:** Comparison of different methods on external datasets.

**Method**	**Acc**	**Specificity**	**AUC**
Kermany et al. (46)	0.934	0.94	0.988
Kaymak and Serener (47)	0.971	0.984	Not mentioned
Concat_Cov2D	**0.974**	**0.991**	**0.983**
Concat_Zeropadding	**0.975**	**0.992**	**0.983**
Concat_Dense	**0.987**	**0.996**	**0.991**

### 4.6. Model interpretability

**Figure 6:**
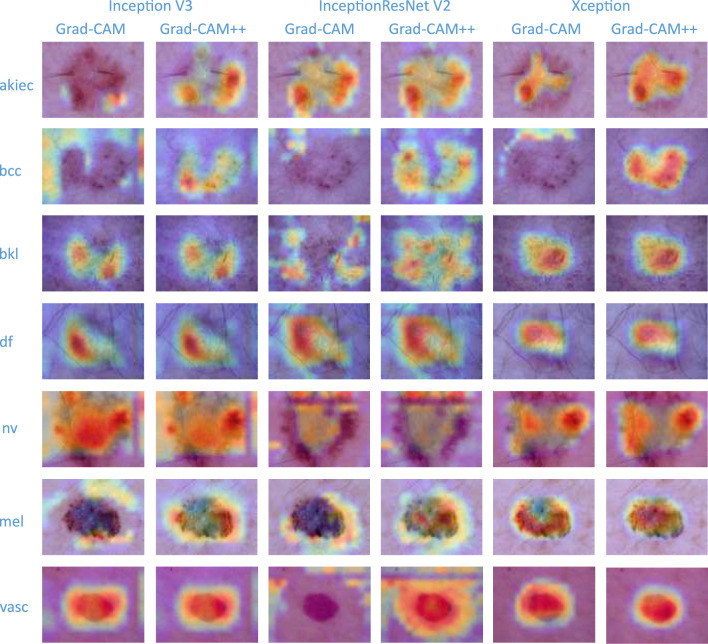
Model visualization.

To verify the interpretable and explainable of the classifier-level fusion network based on the classification layer proposed in this paper, the visualization effect of the sample with the highest prediction probability for each category among the test set samples is shown in Figure 6. In this paper, Grad_CAM (48) and Grad_CAM++ (49) are used as visualization algorithms, and the prediction probability value of the final output category of the test model is used to visualize the fusion of the three base classifiers and the CBAM. To compare the visualization effects of the Grad_CAM and Grad_CAM++ visualization algorithms on the results of this paper and to determine the visualization effect of the final predicted probability value of the model in this paper for the fusion of each base classifier and the attention mechanism, each row in Figure 6 shows that the pictures are all derived from the same sample image. It can be seen from the results that the visualization effects of Grad_CAM++ on the three base classifiers are better than those of Grad_CAM. Grad_CAM++ can display the lesion areas of pigmented skin lesions in a good thermal map. After the image is checked by professional clinicians, the visual part of the image can show that the locations focused on by the model are similar to those yielded by human experience. The visualization effect of Xception shows that the localization area is small and that all results are contained in the lesion area, which is superior to the effects of the other two classifiers (Inception V3 and InceptionResNet V2), thus proving the more interpretable and explainable of the proposed algorithm.

## 5. Conclusion

A fusion network-based detection algorithm for pigmented skin lesions is proposed in this paper. Image preprocessing and image augmentation are carried out before inputting the given dataset into the network, which can solve the problem of low classification accuracy caused by the unbalanced distribution of the original data to a large extent. In this paper, various fusion strategies are used to verify the applicability of the algorithm for pigmented skin lesions. Based on a network performance comparison, we empirically find that the classification effects of the two fusion strategies based on feature-level fusion are not good according to their pigmented skin lesion results. However, the proposed fusion scheme can be applied in other application scenarios and can provide experience guidance for the corresponding model design process. Second, our algorithmic architecture (containing three fusion strategies) only covers single-modal, categorization-oriented methods. However, we also note that multimodal input data are present in medical image analyses, and the corresponding fusion schemes can be studied by extending the current framework (50–52). At the same time, two visualization algorithms are used to apply the color visualization method to make the proposed deep learning model more interpretable and explainable, and the accuracy of the developed algorithm was confirmed by comparing the results with those of related papers. In the future, we plan to test the robustness of the proposed algorithm using a hospital database of actual high definition images of pigmented skin diseases, deploy the algorithm model on servers for physicians in remote areas to diagnose pigmented skin diseases, and apply the three fusion strategies to other more medical application scenarios to validate the advantages of the algorithm.

## Data availability statement

The datasets generated and analysed during the current study are available from the corresponding author upon reasonable request. All deep learning methods are implemented by using TensorFlow (https://tensorflow.google.cn/). The custom script for this study will be available at https://github.com/YHHAZ/NetworkFusion. Correspondence and requests for data materials should be addressed to LC (chlq35@126.com).

## Author contributions

LW and ZA: conceptualization and writing—original draft preparation. LW, ZA, and YL: methodology. ZA, JC, QJ, HC, and QL: writing—review and editing. YL and LC: project administration. JC and QJ: data collection. LW, LC, and YL: funding acquisition. All authors read and agreed to the published version of the manuscript.

## Funding

This work was supported in part by a Wuhan Medical Scientific Research Project grant to LC (WX20B25), in part by a Science and Technology Planning Project of Wuhan grant to LC (2019010701011418), in part by a Research Innovation Fund Project of Jianghan University grant to LW (211051003), and in part by Sinopharm Genomics Technology Co., Ltd. The funders were not involved with the study design; the collection, analysis, or interpretation of data; the writing of this article; or the decision to submit it for publication.

## Conflict of interest

ZA, QL, and YL are employees of Sinopharm Genomics Technology Co., Ltd. The remaining authors declare that the research was conducted in the absence of any commercial or financial relationships that could be construed as potential conflicts of interest.

## Publisher's note

All claims expressed in this article are solely those of the authors and do not necessarily represent those of their affiliated organizations, or those of the publisher, the editors and the reviewers. Any product that may be evaluated in this article, or claim that may be made by its manufacturer, is not guaranteed or endorsed by the publisher.
