# Corrigendum: Detection algorithm for pigmented skin disease based on classifier-level and feature-level fusion

**DOI:** 10.3389/fpubh.2023.1229178

**Published:** 2023-07-18

**Authors:** Li Wan, Zhuang Ai, Jinbo Chen, Qian Jiang, Hongying Chen, Qi Li, Yaping Lu, Liuqing Chen

**Affiliations:** ^1^Dermatology Department, Wuhan No.1 Hospital, Hubei, China; ^2^Dermatology Hospital of Southern Medical University, Guangzhou, China; ^3^Department of Research and Development, Sinopharm Genomics Technology Co., Ltd., Jiangsu, China

**Keywords:** fusion network, pigmented skin disease, attention mechanism, image style transfer, model interpretability

In the published article, there was an error in Figure 1, Figure 2, Figure 6, Table 1, Table 3, Table 4, Table 5, Table 6, Table 7, Algorithm 1 and Algorithm 2. There was an incorrect use of the index “nv”, “mel”, “bcc”, “akiec”, “vasc” and “df” in the original article. The correct index is shown in the “Index mapping” table. “Index” is the index used by the model, “Original index” is the index of published papers, and “Correct index” is the correct index.

**Table:** Index mapping.

**Index**	**Original index**	**Correct index**
0	nv	akiec
1	mel	bcc
2	bkl	bkl
3	bcc	df
4	akiec	nv
5	vasc	mel
6	df	vasc

Corrections have been made to **Detection algorithm for pigmented skin disease based on classifier-level and feature-level fusion**, “*System architecture”*, paragraph two and paragraph three, “*Image preprocessing module*”, “*Image preprocessing and augmentation*”, paragraph two and paragraph four, “*Determination of the experimental parameters*”, *Test results of a single classifier*, paragraph five and paragraph seven. In these paragraphs, “akiec” should be replaced with “nv”.

In the section, **Image preprocessing module**, “*Dataset*”, paragraph one, the corresponding amounts of image data are 6,705, 1,113, 1,099, 514, 327, 142, and 115, respectively.

In the section, **Image preprocessing module**, “*Image preprocessing and augmentation*”, paragraph five, any references to the “original index” should be replaced with “correct index” data.

The authors apologize for these errors and state that this does not change the scientific conclusions of the article in any way. The original article has been updated.

